# Internet of Things, Machine Learning, and Blockchain Technology: Emerging technologies revolutionizing Universal Health Coverage

**DOI:** 10.3389/fpubh.2022.1024203

**Published:** 2022-10-24

**Authors:** Abdulhammed Opeyemi Babatunde, Taofeeq Oluwatosin Togunwa, Olutola Awosiku, Mohd Faizan Siddiqui, Aishat Temitope Rabiu, Abdulqudus Abimbola Akintola, Babatunde Jamiu Dauda, Abdullahi Tunde Aborode

**Affiliations:** ^1^Department of Medicine and Surgery, Faculty of Clinical Sciences, College of Medicine, University of Ibadan, Ibadan, Oyo, Nigeria; ^2^Healthy Africans Platform, Research and Development, Ibadan, Nigeria; ^3^Standing Committee on Medical Education and Research, Federation of African Medical Students' Associations (FAMSA), Ibadan, Nigeria; ^4^One Healthtech Ibadan, Ibadan, Nigeria; ^5^International Medical Faculty, Osh State University, Osh, Kyrgyzstan; ^6^Department of Public Health, Kwara State University, Malete, Kwara, Nigeria

**Keywords:** Universal Health Coverage, Machine Learning, Internet of Things, blockchain, healthcare, Africa

## Introduction

In 2015, all member states at the United Nations General Assembly renewed their commitments toward achieving the Universal Health Coverage by 2030. UHC formed the bedrock of Sustainable Development Goal 3. The goal of the UHC is mainly to provide access to quality health care to everyone without incurring any financial risk. According to the World Health Organization, about 100 million people are pushed to extreme poverty yearly due to out-of-pocket healthcare payments, while about 50% of the world's population lack access to quality health care (1). While some countries of the world like Japan, France, Brazil, and Turkey have successfully achieved this goal, some (Ghana, Indonesia, and Vietnam) are clearly on the course of achieving it by 2030 as planned, but some developing countries like Nigeria, Bangladesh, and Ethiopia are still far from achieving this feat by then (2).

COVID-19's shockwaves have predicted new healthcare concerns worldwide, including severe problems and difficulties in areas like patient consultations, remote monitoring, medical resources, and healthcare staff (3). However, it promoted the development and use of new technologies and innovations in healthcare. These innovations include artificial intelligence, blockchain technology, the Internet of Things, and Big Data and Analytics. These technologies are promising in reducing health expenditure and improving quality and access to healthcare in high, middle, and low-income countries.

In this paper, we discussed our perspective on how the Internet of Things (IoT), Machine Learning (ML), and Blockchain Technology contribute to UHC, especially in developing countries. Internet of Things is a technology that allows the embedding of objects, animals, or people with sensors, software, and other technologies to collect or exchange data on the internet. Machine learning is a subfield of artificial intelligence where computers learn to discover associations and patterns in data using statistical models and apply such associations to predict future results. While blockchain technology allows for the secure management of a shared ledger where transactions are verified and stored on a decentralized network (4).

## Emerging technologies and UHC

### Internet of Things

The Internet of Things (IoT) has recently made significant advancements in healthcare through smart wearables and personal monitoring making it suitable for all age groups and across healthcare specialties (5). The integration of IoT and medical equipment enables healthcare service quality and the tracking of patient progress for individuals who require continuous and real-time medical monitoring and preventative actions. IoT enables the early identification of illnesses and aids in the diagnostic and treatment process in fitness programs, chronic diseases, and elderly patient care. Its usefulness and potential in remote management has been reported during the COVID-19 pandemic (6).

The prevention and management of chronic diseases can be made more affordable with the help of a variety of new technologies. Among them are devices that capture real-time health data when a patient self-administers a therapy. These devices automatically administer treatments or devices that continuously monitor health indicators such as wearable devices, glucose monitors, ECG, amongst other examples (7). A review by Giannakopoulou et al. (8) highlighted latest algorithms that support the cost-effective management of Parkinson's disease using data generated from IoT enabled devices to make accurate predictions through Artificial Intelligence. Many people now utilize mobile applications to manage their different health requirements due to the increasing availability of high-speed Internet and cellphones. The Internet of Medical Things (IoMT) rapidly integrates these devices and smartphone apps with telemedicine and telehealth. Remote monitoring of type-2 diabetes mellitus with intelligent IoMT has been found to be effective in improving medical outcome and lowering burden on both patient and doctor (9). Aside its use in e-Health and health monitoring, IoT is also beneficial in pharmaceutical industry for drug safety, storage and supply chain monitoring (10). There is a growing application and adoption of IoT in healthcare both in high income and low income countries (11).

With all of its benefits, the Internet of Things (IoT) application comes with the possibility of new security possible threats in healthcare systems. Concerns of availability, integrity, and confidentiality are rising (12).

Newer and stronger security standards should be implemented utilizing a resilient method. Moreover, future research on this topic is promising because IoT-based solutions will make it possible to serve the AI algorithms for the prognosis or diagnosis of the disease. They will support innovative healthcare services in general (13). Current and future health IoT solutions should pioneer the way by considering healthcare needs, including usability, interoperability, and security, to have a high impact and success in the healthcare industry shortly. These Industry 4.0 technologies might provide a slew of new ideas and approaches to dealing with medical emergencies locally and globally.

### Machine Learning

The ability of Machine Learning algorithms to see and learn from patterns that are obscure to humans afford them the utilization in mass disease screening, with little or no input from qualified clinical personnel. The use of ML models to support diagnosis have evolved in the last three decades favoring deep learning and clustering facilitated by the adoption of electronic medical records (14). A study carried out in 2020 was able to predict the incidence of Alzheimer's disease in older people by training Machine Learning algorithms on MRI tests using the algorithm called “Support Vector Machines.” The ML algorithm could diagnose the disease with an accuracy, sensitivity, and specificity of 96.12, 94.94, and 98.23%, respectively (15). Catboost is another ML algorithm that screens for anxiety and depression among seafarers, with accuracy and precision of 82.6 and 84.1%, respectively (16). Its use in pediatrics have also been reported mostly in neonatal medicine, psychiatry and neurology mainly due to shortage of experts in these subspecialties (14).

The accurate predictive capability of ML algorithms could potentially reduce the strain and tremendous workload on health workers, with their attention needed only in complicated cases or just for final verification of the algorithm's prediction especially in low-resource settings. However, lack of reliable health data is a major barrier for developing efficient ML models in low and middle income countries.

Aside predictive diagnosis, ML could be used in healthcare insurance system. A study in Rwanda had shown that ML models could predict future out-of-pocket expenditures of households through the sociodemographic variables (17). With households knowing their predicted out-of-pocket expenditure, they can therefore opt for the most appropriate insurance plan to match such spending. This would also allow policymakers to create and implement a range of proper insurance plans to meet these predicted needs, thus optimizing the health insurance system and making the achievement of Universal Health Coverage much more feasible.

Chatbots technology also follows the machine learning can make quality health care delivery independent of a physical hospital building or a compulsory attending clinician. This could help increase access to health care and manage the ever-increasing demand for health services without needing a physical doctor or a visit to a hospital (18). This is achieved by algorithms that provide instant responses to health inquiries, look for generic patterns in specific diseases and diagnose from that place, retrieve and analyze previous health data and set health-related reminders (19).

However, despite the potential of ML, its use in clinical practice is still questioned as it requires more improvement and research (20).

### Blockchain Technology

Blockchain Technology proffers sustainable solutions, mainly to health financing, a significant component of UHC. The emergence of Bitcoin and other cryptocurrencies eliminates third-party financial institutions in global health financing, allowing philanthropists to make donations directly to support the healthcare system quickly and with minimal transaction risks.

Because the blockchain uses the decentralized system, it is open and does not require any permission whatsoever and making it a perfect way forward for healthcare. Blockchain Technology has been used in COVID-19 response such as surveillance, contact tracing, and vaccine monitoring (21). Besides, blockchain technology has also been employed in electronic medical record, IoT devices and supply chain monitoring to secure data effectively (21).

However, there has been a massive demand for blockchain development, and a study conducted by Deloitte revealed that the local industry is searching for an avenue to use blockchain to solve its pressing needs (22).

Immutability, cybersecurity, and interoperability are three peculiar features of blockchain technology that can facilitate adequate data privacy, storage, and management at minimal cost and risk (22). A framework was used to diagnose and treat cancer tumors for some patients remotely using a blockchain model of telemonitoring healthcare and also monitoring of dermatological issues (23). However, it was recommended that guidelines should be put in place for the utilization of contracts, including blockchains which can be used in the validation of data generated at health facilities as well as individual residents.

Blockchain Technology has also been used in geriatrics, management of chronic diseases, biomedical and pharmaceutical industries for research and clinical practice (24).

A survey by Statista 2015 observed an increase in blockchain investment globally, hence, providing opportunities for healthcare financing (25). Despite the significant potential contribution of this technology to health institutions, its usage is still very minimal (Table 1).

**Table 1:** SWOT analysis of blockchain technology.

**Strengths**	**Weaknesses**
• Cost efficiency • Speedy access to medical data • Autonomous • Tamper-proof information sharing	• Less number of software and system vendors • Not much scalable • Lack of storage capacity for a large amount of data
**Opportunities**	**Threats**
• Lower fraud risk in the chain of medical supply • Beneficiaries get more control over the data • Potential for start-ups and forged partnerships in healthcare • The anonymity of data will help in medical research	• Non-standardization • Interoperability issues • Hesitant social adoption of technology • Cultural and trust concerns to adopt blockchain for sensitive data

## Challenges and recommendations

While these emerging technologies look promising in the revolution of UHC, imperfections should also be considered. Some services and resource availability may be disrupted due to the usage of alternative data transmission channels, such as satellite communication in the use of IoT. As a result, an independent and dependable data transmission route is essential to ensure the continuous availability of resources and services (26). Hence, rather than distributing several small IoT networks as timely attachments to global IoT platforms to use their resources and services, provisions should be made for an independent and reliable data transmission channel required for the continuous availability of resources and services (26).

The integration of Blockchain in healthcare to ensure secured automatization of transaction and exchange of information among individuals might be a severely restrictive instrument in which deleting the third-party entity eliminates the only entity capable of preserving human rights (27). Consequently, a decentralization technology might be repurposed into a way of retaining centralized control (27).

Energy usage is rapidly growing due to internet-enabled services and cutting-edge equipment (26). As these technologies largely depend on energy sources for efficient operations, there is the need to create more devices that use less energy and pose almost zero threat to the environment. One of the issues associated with environmental effects is energy consumption by IoT devices (26). Therefore, green technology should produce a more environmentally friendly IoT device with less energy consumption.

The algorithm's capacity of machine learning to generalize new datasets may be hampered since it may leverage potentially untrustworthy unknown cofounders in place of the actual signal (28). In a study by Finlayson et al. (29) it was shown that machine learning algorithms were subject to manipulation by inputs that are purposefully meant to trick them, therefore, posing incapacity outside the domains where they are trained. There is a need for independent datasets that denote future target populations and compare different algorithms while being cautious of signs of potential bias. More work to improve algorithm interpretability and understand human–algorithm interactions will be required for their future adoption and safety, supported by the development of meticulously planned regulatory frameworks.

## Conclusion

Achieving the UHC by 2030 will require an innovative and holistic approach. Emerging technologies in healthcare discussed in this paper have potential in increasing access and reducing cost of healthcare delivery. The shortcomings, and recommendations to leverage the potentials of these technologies for the attainment of the UHC, especially in middle and low-income countries, have been highlighted in this paper.

## Author contributions

AOB and TOT conceptualized and designed the study. TOT, OA, MFS, ATR, AAA, and BJD wrote the first draft of the manuscript. AOB and ATA revised the manuscript. All authors reviewed the final manuscript and agreed to the submission of the manuscript.

## Conflict of interest

The authors declare that the research was conducted in the absence of any commercial or financial relationships that could be construed as a potential conflict of interest.

## Publisher's note

All claims expressed in this article are solely those of the authors and do not necessarily represent those of their affiliated organizations, or those of the publisher, the editors and the reviewers. Any product that may be evaluated in this article, or claim that may be made by its manufacturer, is not guaranteed or endorsed by the publisher.
